# Associations between bedtime eating or drinking, sleep duration and wake after sleep onset: findings from the American time use survey

**DOI:** 10.1017/S0007114521003597

**Published:** 2021-09-13

**Authors:** Su I Iao, Erica C. Jansen, Kerby Shedden, Louise M. O’Brien, Ronald D. Chervin, Kristen L. Knutson, Galit Levi Dunietz

**Affiliations:** 1 Department of Statistics, University of Michigan, Ann Arbor, MI, USA; 2 Department of Nutritional Sciences, School of Public Health, University of Michigan, Ann Arbor, MI, USA; 3 Division of Sleep Medicine, Department of Neurology, University of Michigan, Ann Arbor, MI, USA; 4 Department of Neurology, Northwestern University Feinberg School of Medicine, Chicago, IL, USA

**Keywords:** Mealtime, Sleep, Sleep duration, Wake after sleep onset, Sleep fragmentation

## Abstract

Sleep hygiene recommendations discourage eating before bedtime; however, the impact of mealtime on sleep has been inconsistent. We examined gender-stratified associations between eating or drinking <1, <2 and <3 h before bedtime, sleep duration and wake after sleep onset (WASO >30 min). This study utilised 2003–2018 data from the American Time Use Survey, a nationally representative sample of USA residents aged ≥15 years. Participants recorded weekday/weekend activities during a 24-h period. Age-specific sleep duration and WASO were estimated categorically and continuously. Eating or drinking were identified from all activities recorded <1, <2 and <3 h before bedtime. Mean ± se sleep duration was 8·0 ± 0·006 h, and 6% of participants ate or drank <1 h prior to weekdays bedtime. Overall, eating or drinking <1 h prior to bedtime was associated with longer weekdays sleep duration. Women and men who ate or drank <1 h before bedtime, *v*. those who did not, had 35 min (95% CI (30,39)) and 25 min (95 % CI (21,29)) longer sleep duration, respectively, as well as increased odds of WASO; women (OR=2·03, 95% CI (1·66,2·49)) and men (OR=2·64, 95% CI (2·08,3·36)). As the interval of eating or drinking prior to bedtime expanded, odds of short and long sleep durations and WASO decreased. This population-based data linked eating or drinking <1 h before bedtime to longer sleep duration, but increased WASO. Eating or drinking further from bedtime lowers the odds of short and long sleep duration and WASO. Causal pathways are difficult to discern, though inefficient sleep after late-night eating could increase WASO and trigger compensatory increases in sleep duration.

The American Academy of Sleep Medicine recommends at least 7 h of nocturnal sleep for adults aged 18–60 years to sustain health^(1)^. The contribution of short sleep to poor physical and mental health has been reported consistently^(2–5)^. Further evidence has also associated longer sleep duration, defined as ≥ 9 h, with cardiometabolic morbidities, including diabetes, CVD and mortality^(6)^. Despite these known associations, sleep duration has been declining among USA adults^(7)^, and current prevalence of short sleep duration is estimated at about 35 %^(8)^. Whereas prevalence of short sleep duration among USA adults may be slightly decreasing, prevalence of long sleep duration may be increasing^(9)^.

Wake after sleep onset (WASO) is a key symptom of chronic insomnia^(10)^ and significantly correlates with poor sleep quality^(11)^. Reported by 5–10 % of adults, difficulty to maintain sleep is an insomnia symptom characterised by polysomnography as WASO ≥ 30 min, which is more common among older adults and women^(12)^. Poor mental health and physical morbidity have been associated with WASO in paediatric, pregnant, adult and older adult populations^(13–16)^.

Sleep duration and continuity are influenced by sleep hygiene^(17)^. To promote healthy sleep, sleep hygiene recommendations commonly suggest avoiding electronic screens before bedtime; sleeping in a quiet, dark, cool environment and refraining from using the bedroom for activities other than sleeping or intimacy^(18,19)^. Good sleep hygiene practices also discourage eating or drinking before bed, in particular large or spicy meals, alcohol and caffeinated or carbonated beverages^(20)^. In contrast, light eating before bed is permissible^(18)^. Yet, other than for alcohol and caffeine^(21,22)^, evidence to support dietary recommendations is scarce. Associations of nighttime snacking and irregular or late meal times with poor sleep quality^(23)^, short sleep duration^(24–26)^ and long sleep duration have been suggested^(27)^. However, most of these reports lack a time referent for when nighttime eating occurs in relation to bedtime. A recent study examined the impact of mealtime within 3 h of bedtime on sleep quality and duration in 793 young adults. This study reported positive associations between mealtime and nocturnal awakenings, but not short sleep duration^(23)^. Population-level and gender-specific associations between mealtime and subsequent sleep are rare. Therefore, this study examined overall and gender-specific relationships between weekdays eating or drinking < 1, < 2 and < 3 h before bed, sleep duration and WASO.

## Methods

### Study population: the American time use survey

The American Time Use Survey (ATUS) conducted by the USA Census Bureau is an annual and cross-sectional survey sponsored by the Bureau of Labor Statistics since 2003^(28)^. Except the 2003 sample of *n* 20 720, data from the ATUS have an average sample size of *n* 12 572 (range *n* 9593 to *n* 13 973) in subsequent years. ATUS participants represent the population of USA residents, aged 15 to 85 years. To describe activities along each of the seven weekdays, ATUS was distributed evenly between weekdays and weekends. In an annual phone interview, ATUS participants were asked to report their activities during a 24-h period (04.00 to 04.00 on the interview day) and were randomly selected to report weekdays or weekend activities. However, participants who woke up after 04.00 continued to report activities up to their wake time. Each participant reported either the duration or the start and end time of each activity. The primary analysis focused on the data from 2003 to 2018 and was restricted to weekday respondents (Sunday–Thursday) with nocturnal sleep schedules (*n* 124 239) to examine sleep patterns. Subsequent analysis compared weekend and weekdays sleep patterns.

### Exposure: eating or drinking < 1 h before bedtime

Eating or drinking activities (activity code: 110 101) were identified from all activities recorded within 1, 2 and 3 h prior to bedtime. ATUS respondents were classified into two groups based on the presence or absence of eating or drinking activities < 1, < 2 and < 3 h prior to their primary sleep period. Participants did not record the type of food or drink consumed, only that they had engaged in eating or drinking.

### Primary sleep period

For each ATUS participant, the primary sleep was defined as the period of sleep beginning after 18.00 such that the 5 h following sleep onset contain at least three total hours of sleep. This algorithm identified nocturnal sleep and excluded ATUS participants without primary sleep period or those who reported daytime sleep, a common pattern among women and men who work in shifts.

### Outcome measures

#### Sleep duration on weekdays.

Survey respondents recorded their sleep with an activity code 010 101. Within the primary sleep period, sleep duration was calculated by adding all recorded sleep periods. In this analysis, sleep duration was first considered as a continuous outcome and later as a three-category outcome, i.e., short sleep, sufficient sleep and long sleep, according to the age-specific American Academy of Sleep Medicine recommendations^(29)^. For participants 15–17 years, short sleep, sufficient sleep and long sleep corresponded to < 8 h, 8–10 h and > 10 h, respectively. For those ≥ 18 years, short sleep, sufficient sleep and long sleep were defined as < 7 h, 7–9 h and > 9 h, respectively. Finally, for older adults ≥ 65 years, short sleep, sufficient sleep and long sleep corresponded to < 7 h, 7–8 h and > 8 h, respectively. Total sleep duration ranged between 3 and 14 h. Sleep duration beyond 14 h was reported by 0·2 % of respondents.

#### Self-report Wake After Sleep Onset on weekdays.

Wake after sleep onset is a measure of wakefulness periods that occur after the onset of sleep. WASO was defined for this analysis as any non-sleep activities of ≥ 30 min within the primary sleep period, such that sleep was reported before and after the indicated WASO. This threshold is consistent with the length of sleep-onset insomnia thought to be clinically significant if it recurs on a regular basis^(30)^. We have also computed minutes of WASO as a continuous outcome.

The five most frequent activities that appeared as non-sleep during the primary sleep period were watching television and movies; physical care for household children; washing, dressing and grooming oneself; sleeplessness and eating or drinking (activity codes, respectively: 120 303, 030 101, 010 201, 010 102, 110 101). The total duration of WASO during the primary sleep period was also calculated for those respondents with ≥ 30 min of WASO.

### Potential confounders

Selection of covariates was guided by causal diagrams based on their relevance to eating or drinking behaviours, sleep duration and WASO. The following demographic characteristics were included as potential confounders – gender (men, women), age (15–22, 23–30, 31–50, 51–64, 65+ years), race (White non-Hispanic, Black non-Hispanic, Hispanic and Other), education (less than high school, high school graduate, college graduate, Master’s degree or higher), work status (employed, unemployed and not in labour force), cohabitation (spouse present, unmarried partner present and no partner) and the presence of children < 18 years in the household (yes/no). Age categories were selected to reflect youth, young adulthood, early middle age and older adults, in an effort to compare life stages that may be associated with different amounts and patterns of sleep. The reported day of the week and survey year were also added as covariates to the regression models.

### Statistical analysis

Estimation methods appropriate for complex surveys were used to estimate the frequencies and proportion of ATUS respondents and those who reported eating or drinking < 1 h prior to their bedtime, by socio-demographic characteristics, i.e. gender, age, race, education, work status, cohabitation and the presence of children < 18 years in the household. Appropriate ATUS survey weights were applied in all analyses to yield nationally representative estimates.

Mean weekdays sleep duration and proportion of ATUS respondents who reported WASO were also estimated in relation to these demographic predictors. Bivariate analyses described the proportion of ATUS respondents who reported eating or drinking 1 h prior to bedtime by gender and age and the duration of WASO by gender and age.

Linear and multinomial logistic regression models were used to examine the associations of eating or drinking < 1 h prior to bedtime and sleep duration or WASO, as continuous or categorical outcomes, respectively. Adjusted models controlled for gender, age, race, education, work status, cohabitation, the presence of children < 18 years in the household, day of the week reported and survey year. To evaluate whether gender differences modified the association between eating or drinking prior to bedtime, sleep duration and WASO, we fitted gender–stratified regression models and conducted formal interaction analysis, by adding to our regression models a product term of eating or drinking and gender variables and evaluated its statistical significance as a predictor of sleep measures.

To examine the robustness of our findings, we conducted sensitivity analyses by exclusion of activities within the primary sleep period, unlikely to be related to bedtime eating or drinking. Specifically, within the primary sleep period, we excluded physical care for household children or medical care for household children from WASO. As a second step, we examined the association between bedtime eating or drinking and WASO redefined as a continuous variable. Next, we evaluated the associations between bedtime eating or drinking and sleep duration in respondents who did not report WASO. Exclusion of these respondents allowed examinations of bedtime eating or drinking in relation to continuous sleep duration, i.e. not been affected by WASO. In subsequent analysis of weekdays and weekend differences, we examined the influence of eating or drinking prior to bedtime in relation to sleep duration and WASO on weekends. Finally, we examined eating or drinking activities < 2 and < 3 h prior to bedtime in relation to sleep duration and WASO.

All data analyses procedures were conducted using survey package in R (version 3.5.3) and surveylogistic in SAS (version 9.4).

## Results

**Fig. 1.**
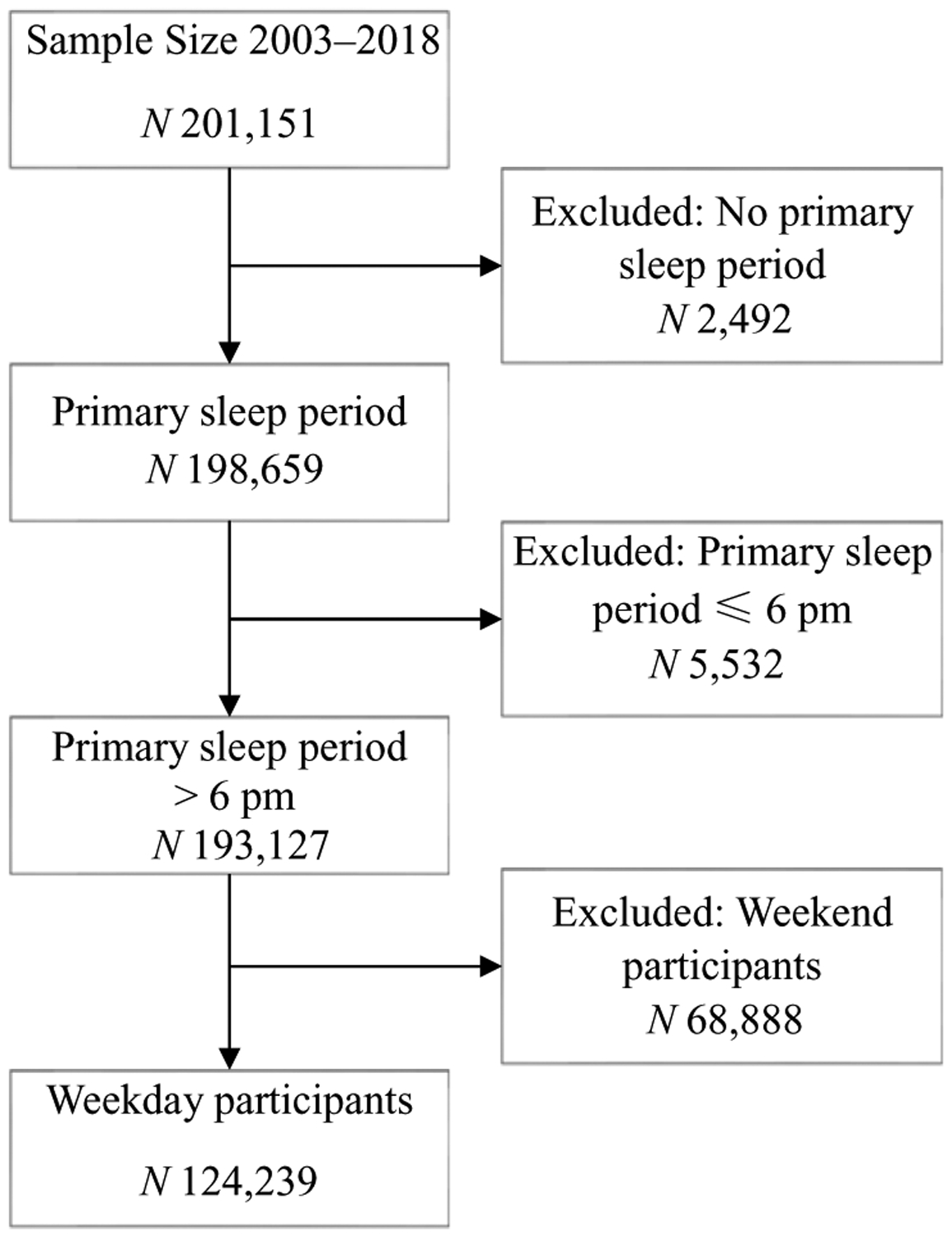
American Time Use Survey (ATUS) participants and final sample size 2003–2018.

More than 201 000 USA residents participated in ATUS surveys between 2003 and 2018. After exclusion of those without primary sleep period, primary sleep period before 18.00 and weekend respondents, the final sample size included 124 239 participants (Fig. 1). Among the 124 239 ATUS survey respondents, 53 % were women, 59 % were aged 50 or younger and the majority (69 %) were white, non-Hispanic. Eating or drinking 1 h prior to bedtime was reported by 6·4 % of survey participants, 5·5 % of women and 7·5 % of men. With increasing age, the proportion of eating or drinking 1 h prior to bedtime declined. The proportion of Americans who ate 1 h prior to bedtime was higher among employed respondents (7·3 %) than among those who were unemployed (6·7 %) or not in labour force (4·8 %) (Table 1).

Mean weekdays sleep duration was somewhat similar for women and men and among racial/ethnic groups. A U-shape trend in mean sleep duration was observed in relation to age, such that those aged 31–64 years had lowest sleep duration (7·8 h). Employed Americans reported shorter weekdays sleep than did the unemployed and those not in the labour force (46 min and 33 min longer, respectively). A higher proportion of women reported WASO than men (2·3 % *v*. 1·4 %), and lowest WASO was reported among respondents in the 15–22 age group (1·1 %) (Table 1).

**Fig. 2.**
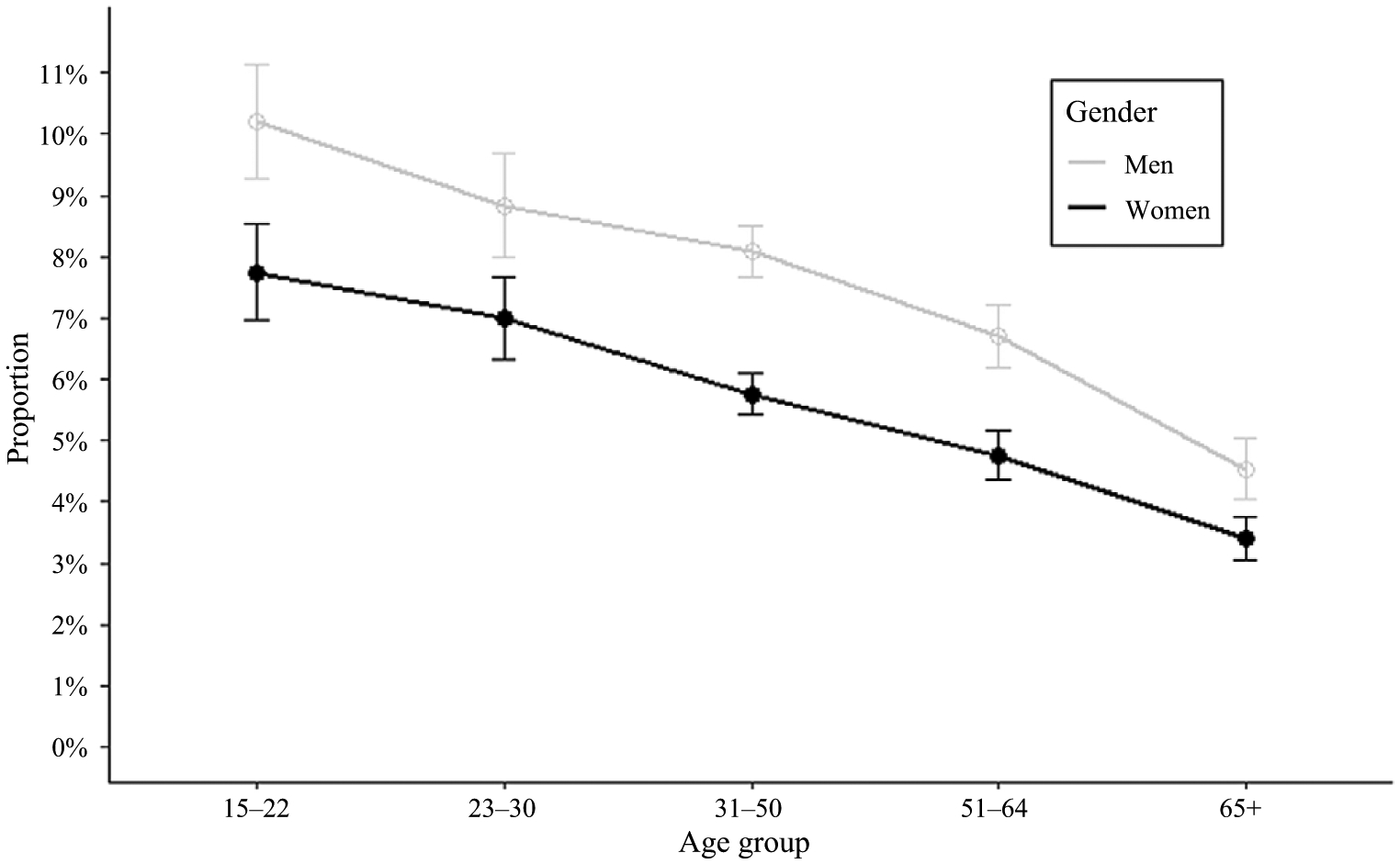
Proportions of Americans eating or drinking < 1 h before bedtime by gender and age.

**Fig. 3.**
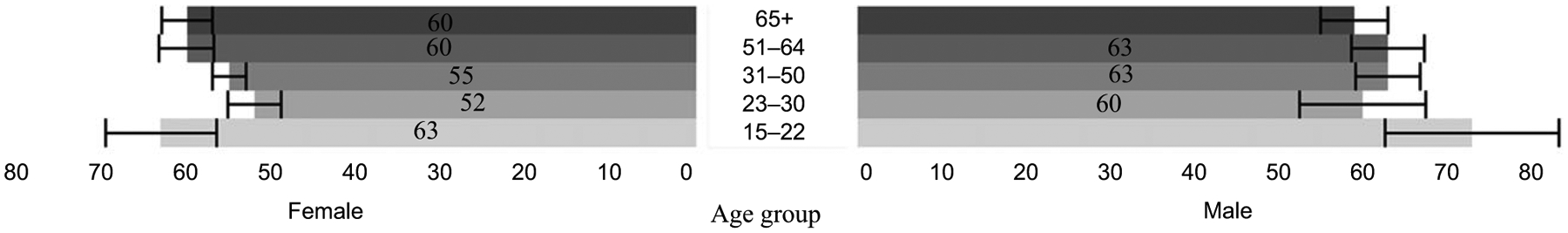
Mean weekdays wake after sleep onset (WASO) minutes by gender and age among those with WASO ≥ 30 min.

The proportion of eating or drinking activities reported < 1 h prior to weekdays bedtime declined along increasing age categories, for both men and women respondents; however, men were more likely to report eating or drinking activities than women along the lifespan (Fig. 2). Among all respondents with reported WASO ≥ 30 min on weekdays, all age groups had on average at least an hour of WASO, except women aged 23–50 years (Fig. 3). The most frequent activities reported during WASO were watching TV and movies, physical care for children, washing, dressing and grooming, sleeplessness and eating or drinking. Description of duration and timing of activities reported by ATUS respondents during weekdays WASO periods, as well as their demographic correlates identified as potential confounders are presented in Table 2.

Linear regression analysis suggested eating or drinking < 1 h prior to bedtime on weekdays was associated with 30 min longer sleep on average (95 % CI 27, 33). The association remained statistically significant after adjusting for gender, age, race, education status, work status, cohabitation and the presence of children < 18 years in the household, day of the week and survey year. An interaction analysis showed significant gender differences in the association between eating or drinking < 1 h prior to bedtime and mean weekdays sleep duration (*P* < 0·001). Unadjusted gender-stratified analyses showed a stronger association between eating < 1 h before bed and weekdays sleep duration among women than men (37 *v*. 26 min longer compared with those who did not eat before bed). In adjusted multinomial logistic regression analysis, eating or drinking < 1 h prior to bedtime was associated with lower odds of short sleep duration on weekdays (odds ratio (OR) = 0·88, 95 % CI: 0·81, 0·94) and increased odds of long sleep (OR = 1·79, 95 % CI: 1·67, 1·91). These results were similar for women and men. Americans who ate < 1 h prior to bedtime were more than twice as likely to report weekdays WASO in comparison with those who did not have eating or drinking activities (OR = 2·26, 95 % CI: 1·93, 2·64). These associations did not vary in a statistically significant manner by gender; OR = 2·03 (95 % CI 1·66, 2·49) for women *v*. OR = 2·64 (95 % 2·08, 3·36) for men. Eating or drinking < 1 h prior to weekdays bedtime was significantly associated with an increase of 13 min of WASO in men. A stratified analyses by weekdays and weekends suggest that women and men who ate or drank < 1 h prior to their bedtime had a longer sleep duration; weekdays – 35 min, 95 % CI (30, 39) and 25 min 95 % CI (21, 29), for women and men, respectively; weekends – 31 min, 95 % CI (25, 38) and 15 min 95 % CI (9, 21) for women and men, respectively. Odds of WASO were higher for women and men who ate or drank < 1 h prior to their bedtime (Table 3).

Sensitivity analyses excluding activities that caused the awakening, e.g. childcare, strengthen the associations between eating or drinking <1 h prior to bedtime and WASO for all respondents, women separately and men separately; OR = 2·47 (2·10, 2·91), 2·33 (1·87, 2·91) and 2·78 (2·18, 3·56), respectively (results not shown).

After exclusion of all ATUS respondents who reported WASO, associations between eating and drinking < 1 h prior to bedtime and long sleep remained significant among both women and men. Associations with short sleep duration were apparent in women and approached statistical significance in men (Table 4).

Associations of eating or drinking < 1, < 2 and < 3 h prior to bedtime with sleep duration and WASO are presented in Table 5. The likelihood of both short and long sleep durations decreased with widening intervals of eating or drinking prior to bedtime. Women who reported eating or drinking < 2 h and < 3 h prior to bedtime had 0·71 and 0·6 odds for short sleep duration in comparison with those who did not eat or drink during that time. Similarly, the odds of short sleep duration among men were 0·75 and 0·66 for eating or drinking < 2 h and < 3 h prior to bedtime, respectively. Overall, as the interval of eating or drinking prior to bedtime lengthen, the odds of short and long sleep durations and WASO decreased (Table 5).

To examine whether this trend persists as intervals between eating or drinking and bedtime continue to expand, we conducted additional analyses with < 4, < 5, < 6 and < 7 h intervals. Indeed, the decreasing associations trend continued with widening intervals; however, the protective effect against *short sleep duration* was most pronounced when eating or drinking was reported < 4 h prior to bedtime (OR = 0·59, 95 % CI (0·57, 0·61), Table 5). Similarly, the odds of WASO and long sleep duration approached a plateau when last eating or drinking activities were < 6 h before bedtime (OR = 1·29, 95 % CI (1·16, 1·45) and OR = 1·09, 95 % CI (1·05, 1·13), respectively).

## Discussion

Data from a large, nationally representative sample show that eating or drinking < 1 h prior to weekday bedtime is associated with 1·8-fold higher odds of long sleep duration (> 9 h) and nearly twofold higher odds of reported WASO ≥ 30 min. Gender differences were observed in sleep duration such that women in comparison with men who ate or drank before bed had a stronger positive association with sleep duration. While eating or drinking < 1 h prior to bedtime was associated with over twofold higher odds of WASO in men and women, this association was more pronounced in men. Alternatively, these findings suggest that refraining from eating or drinking at least 1 h prior to bedtime is protective against WASO. Analysis of WASO as a continuous outcome associated eating or drinking < 1 h prior to bedtime with an increase of 13 min of WASO only in men. As the interval between eating or drinking and bedtime lengthens, the negative impact of eating or drinking prior to bedtime on WASO and long sleep duration is attenuated.

Surprisingly, there are limited investigations on the potential impact of eating or drinking before bedtime on sleep duration, WASO and sleep quality. Prior investigations have primarily examined dietary composition of evening meals in relation to sleep measures and corroborated potential links between evening food intake and subsequent sleep. For example, in toddlers, higher carbohydrate intake in the evening meal was associated with longer sleep duration^(31)^. A study of 45 men with obstructive sleep apnoea and obesity associated higher food intake in the evening period with lower sleep efficiency, lower slow wave sleep, more arousals and a greater apnea-hypopnoea index^(32)^. Among fifty-two men in Brazil, higher nocturnal fat intake (including dinner plus late-night snack) was associated with worse sleep quality, including lower sleep efficiency, longer sleep latency and higher WASO. Women with higher total energetic intake at night, typically 30–60 min before bed, had higher sleep latency and lower sleep efficiency^(25)^, but no associations were observed between nocturnal total energetic intake and sleep among men. These results align with the findings of the present study that the association between eating or drinking before bed and sleep duration was stronger in women. Other studies have shown that snacking patterns, which often include snacking after dinner and up until bedtime, are associated with irregular sleep patterns, both shorter^(24,33)^ and longer than recommended sleep duration for adults (7–9 h)^(27)^.

While eating or drinking closer to bedtime is discouraged, this study demonstrated the negative influence of eating or drinking on sleep duration and WASO at different interval lengths in relation to bedtimes, i.e., < 1, < 2 and < 3 h prior to bedtime. Our results suggest that although associations between eating or drinking and sleep outcomes remained significant, among women and men, regardless of mealtime, effect estimates were attenuated as eating or drinking activities were reported further away from bedtime. This trend persisted as intervals between eating or drinking and bedtime continued expanding to < 4, < 5, < 6 and < 7 h. While the influence of eating or drinking on both short and long sleep duration and WASO was most pronounced when reported < 1 h prior to bedtime, eating or drinking within 4–6 h prior to bedtime confers the highest likelihood for optimal sleep.

The positive association between eating before bedtime and longer sleep duration (> 10 for 15–17 years teens, > 9 for adults or > 8 for older adults) observed in the present study was not in the expected direction. These findings could have several potential interpretations. First, longer self-reported sleep duration could actually indicate efforts to compensate after the experience of more sleep difficulties, such as unsuccessful initiation or maintenance of sleep. The association we report between eating before bed and higher odds of WASO supports this interpretation. Indeed, one of the often-cited reasons for not consuming a large meal before bed or certain types of foods (e.g. spicy food)^(34)^ is that digestive symptoms such as heartburn could disturb sleep^(35)^. Similarly, the consumption of alcohol prior to sleep can adversely affect the quality and continuity of sleep, and caffeine can delay the initiation of sleep^(21)^. Alternatively, the association between eating before bed and longer sleep duration could be attributed to foods that enhance sleepiness. Consumption of certain foods or beverages before bedtime, such as melatonin-enhanced milk, could support longer sleep duration^(34)^. However, the present analysis is unable to disentangle potential food-specific mechanisms as participants were not required to record the type of food consumed. Nonetheless, the possibility remains that no cause-and-effect relationship exists between eating or drinking before bedtime. For example, the associations could reflect clustering of unhealthy habits in both sleep and diet, as these behaviours are often intertwined^(36)^. Further, the association between eating or drinking < 1 h before bedtime and WASO may reflect nocturia, a common sleep disruption, after ingestion of liquids close to bedtime.

This study has several strengths. ATUS is a large, nationally representative survey with over 40 % response rate. Of particular significance, ATUS collects detailed reports of a 24-h period for USA residents along the age continuum, from 15 to 85 years. These detailed data allow the examination of eating or drinking behaviours up to the reported bedtime, as well as sleep timing and waking events during the night. Real-time documentation of eating and sleeping activities avoids recall bias, an inherent limitation of many surveys, particularly for estimated sleep duration. In addition to daily and nocturnal activities, ATUS also collects extensive socio-demographic information now used to adjust for many potentially relevant confounders. Stratification of analysis by weekdays and weekend respondents allowed to identify distinct eating and sleep behaviours as well as to dilute potential confounding related to daylight savings.

However, the present study is not without limitations. As with any cross-sectional survey, the observed associations from one day may not represent habitual sleep and eating behaviours. However, the random assignment of respondents across weekdays and the large sample size allows representation of weekday-specific activities across genders, ages, socio-economic status and regions. Despite the real-time reporting, self-report can introduce inaccuracies. For example, in the estimation of the amount of time spent awake during the night, participants could have rounded their responses. Further inaccuracies in reported sleep duration could result in overestimation or underestimation of sleep duration, based on actual sleep duration. For example, comparisons of objective and self-report sleep duration have suggested that short sleepers (≤ 6 h) were likely to underestimate their sleep duration, while those who slept more than 6 h overestimated the amount of their sleep^(37)^. Nonetheless, we would not expect these measurement errors to be differential with respect to eating behaviours. The present analysis focused on sleep duration and WASO and did not account for sleep timing. Another limitation of ATUS is the absence of data on depressed mood, which can alter food consumption and sleep behaviour. Moreover, the type and amount of food or beverage participants consumed before bed may influence subsequent sleep. In particular, information on alcohol or caffeine consumption was not available. While the ATUS includes a separate module with information about the type of foods consumed by respondents, the timing of those eating activities is missing or they are reported as secondary activity along primary activities unrelated to eating or drinking. Given what is well known about the standard American diet, many of the food items consumed before bed were likely neither healthy nor fresh foods, but rather processed and energy-dense snack items^(38,39)^. The proportion of ATUS respondents who reported WASO seems low, but likely attributed to the self-report method, as polysomnography was not conducted. Finally, data on sleep latency or sleep quality were not reported. Information on specific types of food consumed as well as a more refined examination of sleep characteristics could aid in the formulation of evidence-based recommendations regarding food consumption before bed.

## Conclusion

The present study provides population-level evidence that eating or drinking < 1 h prior to bedtime could have negative impact on WASO but increases sleep duration. Moreover, the further eating or drinking reported from bedtime, the lower odds of short and long sleep duration and WASO observed. These findings suggest that earlier timing of eating or drinking in relation to bedtime – between 4 and 6 h – increases the likelyhood of optimal sleep duration. Future investigations of specific food and beverages in relation to sleep duration and quality could generate nutritional recommendations that benefit sleep health. Although these cross-sectional data cannot prove cause-and-effect relationships, they do help define the magnitude of the potential problem. If eating or drinking before bedtime do make sleep less efficient in essence – longer, with more awakenings – then the magnitude of the impact appears to be substantial. This motivates additional prospective research to assess for any underlying causative pathway.

## Figures and Tables

**Table 1.** Socio-demographic correlates of eating or drinking prior to bedtime, sleep duration and wake after sleep onset (WASO) among 124 239 participants of the American time use survey (Numbers and percentages; mean values and standard deviations)

Socio-demographic predictors	*n*	%	Ate < 1 h before bed, (%)*,†	Mean sleep duration (h)	se	Wake after sleep onset (%)*,†
Gender						
Women	70 125	53	5·5	8·1	0·008	2·3
Men	54 114	47	7·5	7·9	0·009	1·4
Age group, years (y)						
15–22	10 358	12	9·0	8·7	0·022	1·1
23–30	12 908	12	7·9	8·2	0·018	2·2
31–50	48 343	35	6·9	7·8	0·009	1·9
51–64	27 730	23	5·7	7·8	0·012	1·8
65+	24 900	18	3·9	8·2	0·013	2·1
Race/ethnicity						
White, non-Hispanic	85 187	69	5·4	7·9	0·007	1·9
Black, non-Hispanic	16 070	12	7·4	7·9	0·019	2·5
Hispanic	16 704	14	9·6	8·3	0·017	1·3
Other‡	6278	5	8·6	8·0	0·026	1·5
Educational status						
Less than high school	19 308	17	8·4	8·5	0·016	1·6
High school graduate	53 893	45	6·3	8·0	0·009	1·8
College graduate	38 350	29	5·6	7·8	0·009	2·0
Master’s degree or higher	12 688	9	5·8	7·7	0·015	1·9
Work status						
Employed	76 627	62	7·3	7·8	0·007	1·6
Unemployed	5719	5	6·7	8·5	0·030	1·5
Not in labour force	41 893	33	4·8	8·3	0·011	2·3
Cohabitation						
Spouse present	62 863	55	5·9	7·9	0·007	2·1
Unmarried partner present	4041	4	8·4	8·1	0·032	1·4
No spouse or unmarried						
Partner present	57 335	41	6·9	8·2	0·010	1·6
Presence of children < 18 years						
Yes	54 274	40	7·1	8·0	0·009	2·1
No	69 965	60	6·0	8·0	0·008	1·6

* Weighted means and proportions for percentage of respondents who reported WASO ≥ 30 min.

† All bivariate associations are statistically significant, *P* < 0·05.

‡ Other: Asian only, Hawaiian/Pacific Islander only or bi-racial.

**Table 2.** Five frequent weekday activities during wake after sleep onset (WASO) as reported by 2481 American Time Use Survey (ATUS) participants who reported WASO > 30 min on weekdays: duration, timing and demographic correlates (Numbers and percentages; mean values with their standard errors)

	*n*	%	Mean duration (min)	se	Timing	Demographic
Watching television and movies	665	26	59·0	1·38	23:04	Gender: women, 55 % Age: 65+, 30 % Race: white only, 64 % Education: HS graduates, 50 % Employed: 55 % Spouse present: 54 % No child < 18 at home: 70 %
Physical care for household children	519	20	41·3	1·09	00:53 (next day)	Gender: women, 86 % Age: 31–50, 51 % Race: white only, 71 % Education: College graduates, 55 % Employed: 61 % Spouse present: 82 % Child < 18 at home: 100 %
Washing, dressing and grooming oneself	455	19	20·3	0·82	23:30	Gender: women, 61 % Age: 65+, 32 % Race: white only, 65 % Education: HS graduates, 47 % Employed: 51 % Spouse present: 55 % No child < 18 at home: 67 %
Sleeplessness	308	13	49·7	1·76	01:04 (next day)	Gender: women, 67 % Age: 31–50, 32 % Race: white only, 75 % Education: HS graduates, 50 % Employed: 50 % Spouse present: 59 % No child < 18 at home: 62 %
Eating or drinking	173	8	22·6	1·5	23:30	Gender: women, 52 % Age: 51–64, 29 % Race: white only, 62 % Education: HS graduates, 50 % Employed: 51 % Spouse present: 50 % No child < 18 at home: 69 %

HS, High School.

**Table 3.** Associations of eating or drinking prior to bedtime with weekdays and weekend sleep duration and wake after sleep onset (WASO) among 124 239 participants of the American time use survey (Odds ratio and 95 % confidence intervals)

	Sleep duration*		Short sleep†,‡		Long sleep†,‡		WASO*		WASO ≥ 30 min §,∥	
	Beta	95 % CI	Odds ratio	95 % CI	Odds ratio	95 % CI	Odds ratio	95 % CI	Odds ratio	95 % CI
All respondents										
Ate < 1 h before bedtime										
Crude model	30	27, 33*	0·90	0·83, 0·96*	1·55	1·45, 1·65*	8·24	4·54, 11·95*	2·00	1·72, 2·33*
Adjusted model¶										
Weekdays	29	26, 32*	0·88	0·81, 0·94*	1·79	1·67, 1·91*	7·73	4·07, 11·39*	2·26	1·93, 2·64*
Weekend	23	18, 27*	1·03	0·92, 1·15	1·49	1·37, 1·61*	7·04	1·72, 12·35*	1·74	1·39, 2·18*
Women										
Ate < 1 h before bedtime										
Crude model	37	32, 41*	0·85	0·76, 0·95*	1·60	1·46, 1·75*	4·77	0·38, 9·17*	1·94	1·58, 2·37*
Adjusted model¶										
Weekdays	35	30, 39*	0·84	0·75, 0·94*	1·80	1·63, 1·97*	4·15	−0·25, 8·54	2·03	1·66, 2·49*
Weekend	31	25, 38*	0·98	0·83, 1·16	1·62	1·43, 1·82*	7·77	1·25, 14·30*	1·89	1·41, 2·53*
Men										
Ate < 1 h before bedtime										
Crude model	26	22, 30*	0·91	0·83, 1·01	1·55	1·42, 1·69*	12·79	6·48, 19·10*	2·32	1·83, 2·93*
Adjusted model¶										
Weekdays	25	21, 29*	0·90	0·82, 0·99*	1·78	1·62, 1·95*	13·28	7·19, 19·37*	2·64	2·08, 3·36*
Weekend	15	9, 21*	1·07	0·91, 1·25	1·38	1·23, 1·55*	6·76	−2·08, 15·60	1·56	1·09, 2·23*

* From a linear regression model with sleep duration or WASO in minutes as the outcome and eating or drinking < 1 h before bed as a dichotomous predictor.

† From a multinomial logistic model with short sleep duration, sufficient sleep duration and long sleep duration as categorical outcomes (sufficient sleep was the reference) and eating or drinking < 1 h before bed as a dichotomous predictor.

‡ For participants 15–17 years, short sleep, sufficient sleep and long sleep corresponded to < 8 h, 8–10 h and > 10 h, respectively. For those ≥ 18 years, short sleep, sufficient sleep and long sleep were defined as < 7 h, 7–9 h and > 9 h, respectively. Finally, for older adults ≥ 65 years, short sleep, sufficient sleep and long sleep corresponded to < 7 h, 7–8 h and > 8 h, respectively. (Reference: Hirshkowitz M et al. (2015). National Sleep Foundation’s sleep time duration recommendations: methodology and results summary. Sleep Health 1, 40–43.)

§ From a logistic regression model with WASO ≥ 30 min as the dichotomous outcome and eating or drinking < 1 h before bed as a dichotomous predictor.

∥ WASO = wake after sleep onset ≥ 30 min.

¶ Adjusted for gender, age, race, education, work status, cohabitation, the presence of children < 18 years in the household, day of the week and survey year.

**Table 4.** Associations of eating or drinking prior to bedtime with weekdays sleep duration among participants of the American time use survey who did not report wake after sleep onset (WASO) (Odds ratio and 95 % confidence intervals)

	Sleep duration†		Short sleep‡		Long sleep‡	
	Beta	95 % CI	Odds ratio	95 % CI	Odds ratio	95 % CI
All respondents						
Ate < 1 h before bedtime						
Crude model	30	27, 33*	0·90	0·84, 0·97*	1·54	1·44, 1·64*
Adjusted model§	29	26, 32*	0·88	0·81, 0·95*	1·77	1·66, 1·90*
Women						
Ate < 1 h before bedtime						
Crude model	37	32, 42*	0·85	0·76, 0·95*	1·59	1·45, 1·74*
Adjusted model§	35	30, 39*	0·84	0·75, 0·94*	1·79	1·63, 1·97*
Men						
Ate < 1 h before bedtime						
Crude model	25	21, 30*	0·92	0·84, 1·02	1·54	1·41, 1·68*
Adjusted model§	24	20, 28*	0·91	0·82, 1·00	1·76	1·61, 1·94*

* Statistically significant at *P* < 0·05; WASO, wake after sleep onset.

† From a linear regression model with sleep duration in minutes as the outcome and eating or drinking < 1 h before bed as a dichotomous predictor.

‡ From a multinomial logistic model with short sleep duration, sufficient sleep duration and long sleep duration as categorical outcomes (sufficient sleep was the reference) and eating or drinking < 1 h before bed as a dichotomous predictor.

§ Adjusted for gender, age, race, education, work status, cohabitation, the presence of children < 18 years in the household, day of the week and survey year.

**Table 5.** Timing of eating or drinking prior to bedtime in relation to sleep duration and wake after sleep onset (WASO) among 124 239 participants of the American time use survey (Odds ratio and 95 % confidence intervals)

	Sleep duration†		Short sleep‡		Long sleep‡		WASO ≥ 30 min§	
	Beta	95 % CI	Odds ratio	95 % CI	Odds ratio	95 % CI	Odds ratio	95 % CI
All respondents								
Timing of eating or drinking								
Crude model								
<1 hour	30	27, 33*	0·90	0·83, 0·96*	1·55	1·45, 1·65*	2·00	1·72, 2·33*
<2 hours	28	26, 30*	0·74	0·70, 0·77*	1·29	1·24, 1·35*	1·81	1·62, 2·02*
<3 hours	28	26, 29*	0·63	0·61, 0·65*	1·17	1·13, 1·21*	1·65	1·50, 1·81*
Adjusted model∥								
<1 hour	29	26, 32*	0·88	0·81, 0·94*	1·79	1·67, 1·91*	2·26	1·93, 2·64*
<2 hours	30	28, 31*	0·73	0·70, 0·77*	1·60	1·51, 1·65*	2·01	1·80, 2·25*
<3 hours	31	30, 33*	0·63	0·61, 0·66*	1·48	1·43, 1·54*	1·80	1·63, 1·99*
<4 hours	30	29, 32*	0·59	0·57, 0·61*	1·33	1·28, 1·37*	1·55	1·40, 1·71*
<5 hours	26	25, 28*	0·60	0·58, 0·62*	1·17	1·13, 1·21*	1·39	1·26, 1·55*
<6 hours	21	19, 22*	0·66	0·63, 0·68*	1·09	1·05, 1·13*	1·29	1·16, 1·45*
<7 hours	16	14, 18*	0·72	0·69, 0·75*	1·06	1·01, 1·10*	1·28	1·14, 1·45*
Women								
Timing of eating or drinking								
Crude model								
<1 hour	37	32, 41*	0·85	0·76, 0·95*	1·60	1·46, 1·75*	1·94	1·58, 2·37*
<2 hours	32	29, 34*	0·70	0·66, 0·75*	1·32	1·24, 1·39*	1·66	1·45, 1·92*
<3 hours	32	30, 34*	0·59	0·56, 0·62*	1·22	1·16, 1·27*	1·60	1·42, 1·80*
Adjusted model∥								
<1 hour	35	30, 39*	0·84	0·75, 0·94*	1·80	1·63, 1·97*	2·03	1·66, 2·49*
<2 hours	32	30, 35*	0·71	0·66, 0·76*	1·59	1·49, 1·69*	1·74	1·50, 2·01*
<3 hours	34	33, 36*	0·60	0·57, 0·64*	1·54	1·47, 1·61*	1·66	1·47, 1·87*
Men								
Timing of eating or drinking								
Crude model								
<1 hour	26	22, 30*	0·91	0·83, 1·01	1·55	1·42, 1·69*	2·32	1·83, 2·93*
<2 hours	26	23, 29*	0·76	0·71, 0·81*	1·30	1·22, 1·38*	2·24	1·88, 2·68*
<3 hours	24	22, 26*	0·66	0·63, 0·70*	1·13	1·07, 1·18*	1·86	1·57, 2·19*
Adjusted model∥								
<1 hour	25	21, 29*	0·90	0·82, 0·99*	1·78	1·62, 1·95*	2·64	2·08, 3·36*
<2 hours	27	24, 30*	0·75	0·70, 0·81*	1·57	1·47, 1·68*	2·56	2·13, 3·01*
<3 hours	28	26, 30*	0·66	0·62, 0·69*	1·41	1·34, 1·49*	2·09	1·77, 2·48*

* Statistically significant at *P* < 0·05; WASO, wake after sleep onset.

† From a linear regression model with sleep duration in minutes as the outcome and timing of eating or drinking before bedtime as a dichotomous predictor, < 1 h, < 2 h, < 3 h and so forth.

‡ From a multinomial logistic model with short sleep duration, sufficient sleep duration and long sleep duration as categorical outcomes (sufficient sleep was the reference) and timing of eating or drinking before bedtime as a dichotomous predictor, < 1 h, < 2 h, < 3 h and so forth.

§ From a logistic regression model with WASO ≥ 30 min as the dichotomous outcome and timing of eating or drinking before bedtime as a dichotomous predictor, < 1 h, < 2 h, < 3 h and so forth.

∥ Adjusted for gender, age, race, education, work status, cohabitation, the presence of children < 18 years in the household, day of the week and survey year.

## Abbreviations:

ATUS: American Time Use Survey
WASO: wake after sleep onset
